# Exploring the Role of Mesenchymal Stem Cell–Derived Exosomes in Diabetic and Chemotherapy-Induced Peripheral Neuropathy

**DOI:** 10.1007/s12035-024-03916-z

**Published:** 2024-01-22

**Authors:** Lamiaa A. Ahmed, Khaled F. Al-Massri

**Affiliations:** 1 Department of Pharmacology and Toxicology, Faculty of Pharmacy, Cairo University, Kasr El Aini St, Cairo, 11562 Egypt; 2 Department of Pharmacy and Biotechnology, Faculty of Medicine and Health Sciences, University of Palestine, Gaza, Palestine

**Keywords:** Chemotherapy, Diabetes, Exosomes, Mesenchymal stem cells, Peripheral neuropathy

## Abstract

Diabetic and chemotherapy-induced peripheral neuropathies are known for long-term complications that are associated with uncontrolled hyperglycemia and cancer treatment, respectively. Peripheral neuropathy often requires long-term therapy and could persist after treatment provoking detrimental effects on the patient’s quality of life. Despite continuous drug discoveries, development of efficient therapies is still needed for the significant management of diabetic and chemotherapy-induced peripheral neuropathy. Exosomes are nanosized extracellular vesicles that show great promise recently in tissue regeneration and injury repair compared to their parent stem cells. Herein, we provided a summary for the use of mesenchymal stem cell–derived exosomes in diabetic and chemotherapy-induced peripheral neuropathy in addition to recent advancements and ways proposed for the enhancement of their efficacy in these diseases.

**Graphical abstract:**

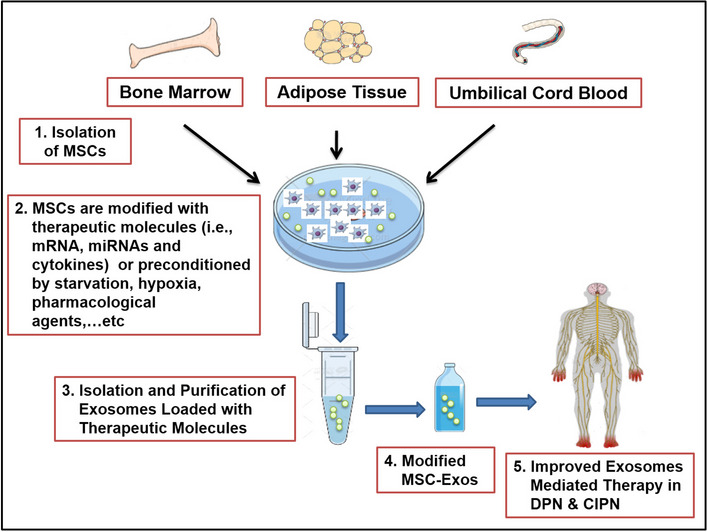

## Introduction

Understanding the painful neuropathic disorders and mechanisms of current therapies is crucial for the recommendation of effective management strategies [1]. Peripheral neuropathy (PN) is associated with several diseases including diabetes, infections, autoimmunity, malignancy, and several metabolic diseases [2]. The most common causes of PN are diabetes and treatment with chemotherapy [3] with prevalence exceeding 50% in people with diabetes [4] and approximately 20 to 85% in patients treated with chemotherapy [5]. The exact mechanisms and pathogenesis of diabetic peripheral neuropathy (DPN) and chemotherapy-induced peripheral neuropathy (CIPN) remain unclear, although it has been suggested that both share mitochondrial dysfunction as a common pathogenic mechanism [6].

Diabetic sensorimotor polyneuropathy (DSPN) is one of the known type of diabetic neuropathy which demonstrates significant morbidity, affecting the quality of the patient’s life and increasing healthcare costs [7]. The progress of DSPN could be related mainly to defect in the antioxidant defense mechanisms. Other underlying mechanisms include changes in blood flow supplying the peripheral nerves, autoimmune and metabolic disorders leading to glial cell activation, changes in ion channel expression including sodium and calcium channels, and increased thalamic vascularity in addition to imbalance of stimulatory/inhibitory descending pathways with the involvement of central pain mechanisms [7, 8]. Other risk factors that contribute to DPN include old age, prolonged duration of uncontrolled diabetes, smoking, and drinking alcohol [9].

The clinical manifestations of DPN are comparable to those of CIPN. CIPN is a prominent complication associated with the long-term toxicity of chemotherapies including platinum-based drugs (cisplatin, carboplatin, and oxaliplatin), taxanes (paclitaxel and docetaxel), vincristine, and eribulin [10]. CIPN may persist for several years even after the cessation of chemotherapy causing disability in cancer survivors and seriously affecting their quality of life [11–13]. CIPN demonstrates sensory symptoms including pain, numbness, and tingling. However, some patients may have difficulties in fine motor coordination and sensory ataxia in addition to autonomic dysfunction [14]. The pathophysiological processes of CIPN are multi-factorial and have not been fully explained but differ among various classes of chemotherapeutic agents [15]. These processes involve oxidative stress, mitochondrial damage, apoptosis, altered ion channel activity, microtubular damage, axon degeneration and demyelination, and neuroinflammation [15, 16].

Several treatments have been proposed by international guidelines for controlling only symptoms of PN irrespective of the underlying cause. First-line therapies mostly include serotonin–noradrenaline reuptake inhibitors, tricyclic antidepressants, and anticonvulsants which act on sodium and calcium channels. Other treatments include the use of opioids in addition to topical agents such as lidocaine and capsaicin [9]. All traditional therapies reveal partial symptomatic relief of DPN and CIPN which reverses upon discontinuation in addition to having dose-limiting potential side effects [16]. These side effects often hinder the therapeutic options in clinical practice making the current treatments for DPN and CIPN suboptimal with urgent need for efficacious therapeutic strategies.

Mesenchymal stem cells (MSCs) are multipotent non-hematopoietic cells which show multiple advantages over other types of stem cells. MSCs can be readily isolated from different sources like adipose tissue, peripheral blood, and cord blood with minimally invasive approaches [17] and is known to be self-replicated to many passages [18]. MSCs have been widely considered a promising cell therapy for management of various neurological disorders [19, 20] with evidence of their efficacy in DPN [21–23] and CIPN [24, 25]. MSCs promote PN repair primarily via their paracrine effects with the secretion of angiogenic, anti-inflammatory, and neurotrophic factors [26–28]. However, MSC transplantation is compromised by prolonged induction period as well as possible risks of immunogenicity, tumor formation, and microcirculatory obstruction [29–31].

Exosomes are endosomal-origin membranous nanovesicles with a diameter ranging from approximately 50 nm to 100 nm [32]. Exosomes are released mostly by all cell types and play a pivotal role in communication between cells by acting as biological transporters. Exosomes contain functional mRNAs, microRNAs (miRNAs), proteins, and lipids [33]. Current studies have shown that MSC-derived exosomes (MSC-exos) are with similar functions to their parent cells. However, unlike MSCs, exosomes possess lower immunogenicity because they possess fewer membrane-bound proteins [34] and have a reduced risk of microvasculature occlusion [35]. Exosomes can also cross biological barriers including the blood-brain barrier because of their nanoscale structures and their membrane composition [36, 37]. Moreover, compared to their parent cells, exosomes can be stored easier and without the use of potentially toxic cryopreservatives for long term, reducing their toxic side effects upon application [38].

The therapeutic effect of MSC-exos has been demonstrated in a variety of diseases, including tumors [39], neurodegenerative diseases [40], and cardiovascular [41] and cerebrovascular [42] disorders. Importantly, several studies demonstrated the therapeutic effect of MSC-exos in DPN. However, the impact of MSC-exos in CIPN is still limited, and it is important to highlight on this crucial type of PN to encourage more research to be done in this field. Future researches are also required to fully elucidate in detail the mechanism of actions of MSC-exos in different models of PN. Of note, a series of challenges and difficulties need to be overcome to verify and enhance the therapeutic efficacy of the derived exosomes and to promote their clinical application.

## Role of MSCs in PN

To develop more effective therapies to neuropathic pain, the potential of stem cells has been assessed in various stages of sensory neuropathy [25, 43] where stem cell therapy demonstrates beneficial outcomes experimentally in sensory neuropathy associated with diabetes [23], spinal cord injury (SCI) [44, 45], and ligation of sciatic nerve [46]. These outcomes depend mainly on the neuroregenerative and neuroprotective potentials of stem cells as demonstrated previously [47–50]. More importantly, the analgesic actions of MSCs have been revealed both experimentally [22, 51] and in clinical trials [52, 53], highlighting the efficacy of this therapy in the management of neuropathic pain. In this context, MSCs have been the most investigated type due to their wide therapeutic potential, minor risk of tumorigenesis, and ease of isolation and expansion in vitro [54]. However, the mechanisms involved in the therapeutic effects of MSCs have not yet been fully verified, but most probably, multiple mechanisms are included, clarifying the broad biological properties of these cells [22, 23, 46, 51].

MSCs promote the repair and modulate the injured neuronal environment through secretion of trophic, anti-inflammatory, and antiapoptotic factors which support angiogenesis, immunomodulation, remyelination, and axonal growth in addition to providing protection from high apoptotic cell death [28]. Through the support of angiogenesis, MSCs augment the microcirculation supplying the peripheral nerves where the impairment of vascular supply has been significantly revealed in DN. MSCs also could be differentiated into endothelial cells and neurons in addition to the secretion of important factors, like angiogenin, basic fibroblast growth factor, vascular endothelial growth factor-A , and nerve growth factor [55, 56], which are crucial to both vascular and neuronal tissue health. Obviously, adipose-derived MSCs secrete increased levels of various angiogenic growth factors both directly and indirectly accelerating the healing of diabetic wounds in rats [57]. MSCs also provide a potential therapy for CIPN through suppressing the cascades of neuronal oxidative stress, inflammation, and apoptosis and promoting axonal repair and regeneration [16].

## Role of Exosomes in PN

A number of in vivo and in vitro studies have shown the ability of exosomes to regulate immune responses, promote angiogenesis, and regenerate damaged tissue [58]. Exosomes are endosomal membranous nanovesicles which represent the main and vital components of small extracellular vesicles (< 100 nm) [59–61]. Exosomes could promote the communication between cells by transferring cargo genomic materials including miRNAs as well as proteins and lipids [62, 63]. Exosomes could be used for the management of DPN where the administration of exosomes intravenously from healthy Schwann cells remarkably improved PN in a model of diabetes in mice [64]. However, there is a limitation of studies using exosomes in CIPN. Obviously, exosome cargo shows the same properties as their parent cells [65, 66] where exosomes acquired from mesenchymal stromal cells contain peptides and miRNAs that promote neuronal repair and function [63]. MSC-exos have been found to be internalized by distal axons of cortical neurons with the mediation of axonal growth even under inhibitory conditions [67]. Moreover, exosomes derived from cerebral endothelial cells (CEC-exos) when used in combination with a platinum drug significantly decreased CIPN and increased the anti-tumor effect of oxaliplatin in mice with ovarian cancer. After being internalized into neuronal fibers, the intravenously administered CEC-exos modified miRNA and protein networks of sciatic nerve and thus reduced the toxicity of peripheral nerves [68]. A previous study also revealed that the application of bone marrow mesenchymal stem cell-derived exosomes (BM-MSC-exos) reduced the expression of tumor necrosis factor α (TNF-α) and transforming growth factor β and increased the levels of interleukin (IL)-10 and arginase 1 in a mouse model of DPN, indicating a decrease in macrophages M1/M2 ratio. These outcomes could be mediated through the polarization of macrophages M2 with subsequent inhibition of the expression of pro-inflammatory genes and improvement of neurovascular function [69]. Additionally, exosomes carrying miR-181c-5p alleviated chronic constriction injury–induced neuropathic pain through inhibition of neuropathic inflammation [70].

## Application of MSC-exos in DPN

**Fig. 1:**
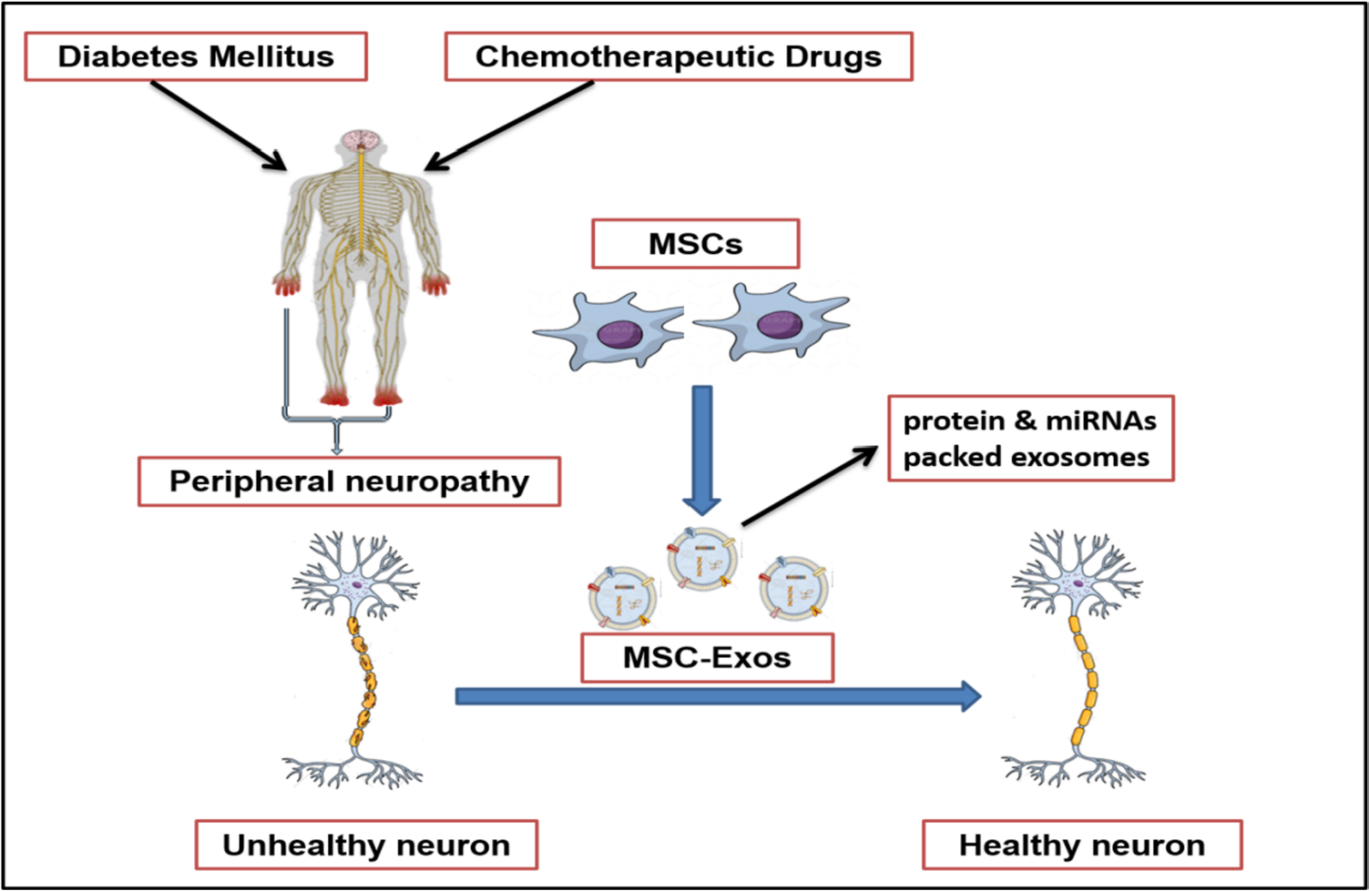
Role of mesenchymal stem cell–derived exosomes in diabetic and chemotherapy-induced peripheral neuropathy

Exosomes play a pivotal role in the communication between cells by transporting biological molecules where they reveal low immunogenicity and the ability to cross the blood-brain barrier. The therapeutic effect of MSC-exos has been demonstrated in preclinical studies of different nervous system diseases [33, 71, 72] with significant impact in DPN (Fig. 1).

For example, the delivery of MSC-exos was shown to inhibit the inflammatory response and promote the neurovascular remodeling and functional recovery of DPN in diabetic mice [69]. MSC-exos also ameliorated DN by autophagy induction through the mammalian target of rapamycin (mTOR) signaling pathway in a rat model of streptozotocin-induced diabetes mellitus [73]. Moreover, exosomes derived from Schwann cells ameliorated PN in type 2 diabetic mice [64]. Furthermore, exosomes when used as biologic vehicles of miR-146a effectively enhanced the therapeutic action of MSCs in diabetic mice, providing a new therapeutic approach for the management of DPN [74]. Table 1 shows some studies on MSC-exos in the treatment of DPN.

**Table 1:** Examples for some studies on mesenchymal stem cell–derived exosomes in the treatment of diabetic peripheral neuropathy

Title of study	Outcomes	Reference
Mesenchymal stromal cell–derived exosomes ameliorate peripheral neuropathy in a mouse model of diabetes	Reduction of the inflammatory response and promotion of neurovascular remodeling and functional recovery of DPN in diabetic mice	[69]
Treatment of diabetic peripheral neuropathy with engineered mesenchymal stromal cell–derived exosomes enriched with microRNA-146a provides amplified therapeutic efficacy	Enhancement of the therapeutic activity of MSCs in diabetic mice through the use of exosomes as biologic vehicles of miR-146a, providing a novel therapeutic strategy for improvement of neurovascular remodeling and functional recovery of DPN	[74]
Transplantation of engineered exosomes derived from bone marrow mesenchymal stromal cells ameliorates diabetic peripheral neuropathy under electrical stimulation	Providing a new treatment approach for DPN using BM-MSC-exos and conducting system such as polypyrrole nanoparticles and exogenous electrical stimulation	[75]
Evaluation of polymeric-aligned NGCs and exosomes in nerve injury models in diabetic peripheral neuropathy condition	Enhancement of sciatic nerve regeneration using nerve conduits and NGCs along with exosomes	[76]

*BM-MSCs* bone marrow mesenchymal stem cells, *BM-MSC-exos* bone marrow mesenchymal stem cell–derived exosomes, *DPN* diabetic peripheral neuropathy, *MSCs* mesenchymal stem cells, *NGCs* nerve guidance channels

## Application of MSC-exos in CIPN

Therapeutic approaches for the management of CIPN are limited and have been based on evidences provided from other types of neuropathies including diabetes and post-herpetic infections [77, 78]. No currently available drugs are considered fully effective in the clinical management of painful neuropathy associated with chemotherapy. However, the choice depends mostly on the use of drugs that reduce neural excitability, such as gabapentin, which still has low efficacy in reducing pain [79, 80].

**Fig. 2:**
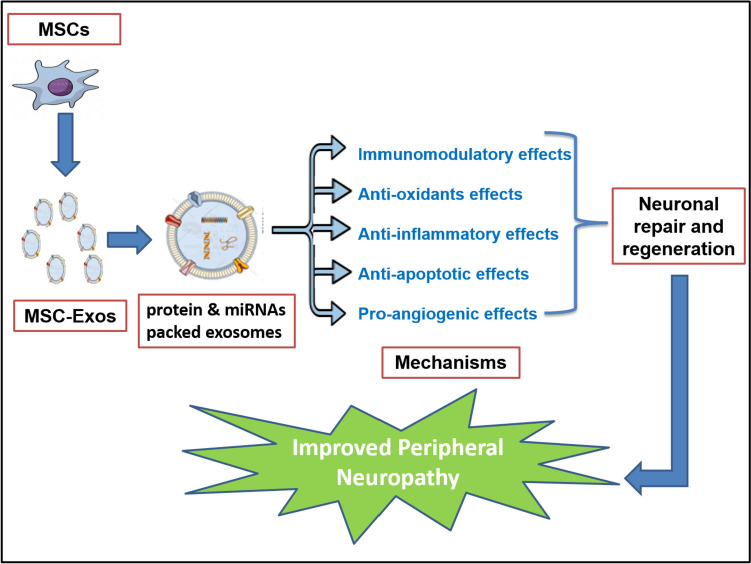
Several beneficial mechanisms of mesenchymal stem cell–derived exosomes in diabetic and chemotherapy-induced peripheral neuropathy

Several mechanisms have been proposed to mediate the beneficial actions of MSC-exos including the secretion of angiogenic and neurotrophic factors, enhanced production of immunosuppressive factors including IL-10, and healthy mitochondrial transfer from MSCs to the damaged neurons [81–83]. Notably, several reports have demonstrated that exosomes derived from BM-MSCs contain a reparative cargo with various cytokines, neurotrophic factors, and miRNA, which mediate significant anti-inflammatory and anti-apoptotic actions with well-recognized immunomodulatory properties (Fig. 2). BM-MSCs and BM-MSC-exos can modulate microglia and astrocyte reactivity, thereby promoting neuro-regeneration [84, 85]. However, assessment of the detailed molecular and cellular mechanisms that affect neural microenvironment after these therapies still requires more studies.

Experimentally, intrathecal administration of MSC-exos reduced mechanical and thermal hypersensitivity in L5/6 spinal nerve ligation neuropathic pain rat model. The anti-inflammatory actions of these exosomes have been demonstrated through diminishing c-fos, 2′,3′-cyclic nucleotide 3′-phosphodiesterase (CNPase), glial fibrillary acidic protein, ionized calcium–binding adaptor molecule 1 (Iba1), TNF-α, and IL-1β levels in addition to enhancing IL10, brain-derived neurotrophic factor (BDNF), and glial cell line–derived neurotrophic factor levels [86]. CEC-exos also reduced CIPN and enhanced the anti-tumor efficacy of platinum drugs [87]. Importantly, a recent study demonstrated that cannabidiol incorporated in extracellular vesicles derived from human umbilical cord MSCs had the ability to alleviate mechanical and thermal pain sensitivities in paclitaxel-induced PN. These outcomes were associated with mitochondrial protection in neuronal cells via activating 5HT1A receptors and AMP-activated protein kinase (AMPK) pathway [88]. In another study, BM-MSC-exos loaded with reduced graphene oxide (rGO), gelatin-methacryloyl (GelMA), and polycaprolactone (PCL) to create an rGO-GelMA-PCL nerve conduit revealed a superior ability to alleviate sciatic nerve injury via increasing the number of newly formed vessels as well as promoting peripheral nerve regeneration and function recovery [89]. Thus, despite being limited, the results of published studies give the hope for the possible significant impact of MSC-exos application in CIPN.

## Enhancement of the Therapeutic Effects of MSC-exos in Different Models of Neuropathy

MSC-exos exert clear therapeutic effects in various diseases. Obviously, exosome properties vary depending on the status of MSCs from which they are derived where MSC status is modified in response to external stimuli. Therefore, several studies have investigated whether preconditioning MSCs could enhance the therapeutic activities of their derived exosomes. Preconditioning of MSCs with cytokines, hypoxia, and chemicals improve their immunosuppressive, immunomodulatory, and regenerative effects [90]. Moreover, genetic and cell surface modification can enhance the therapeutic efficacy of exosomes [91].

## Different Preconditioning Strategies

### Preconditioning of MSCs with Hypoxia

The administration of MSC-exos subjected to hypoxia preconditioning could enhance the neurologic functions by specific biomolecule transport to the recipient damaged cells [41]. For example, exosomes derived from the exposure of MSCs to hypoxia preconditioning restored the synaptic dysfunction and reduced the inflammatory responses through the regulation of miR-21 secretion and thus improvement of the learning and memory capabilities of APP/PS1 mice [41]. Hypoxia preconditioning represents one of the most effective approaches to enhance the therapeutic effects of MSC-exos. A possible underlying mechanism could be through enhanced exosomal miR-216a-5p secretion which directs the shifting of microglia from the pro-inflammatory M1 phenotype to the anti-inflammatory M2 phenotype and thus suppressing toll-like receptors (TLRs)/nuclear factor kappa B (NF-κB) and activating phosphatidylinositol 3-kinase (PI3K)/protein kinase B (PKB) signaling pathways [92]. Another study indicated that human umbilical cord mesenchymal stem cell (HUCMSC)-derived exosomes could improve the viability and migration of olfactory ensheathing cells in hypoxic conditions by activating BDNF signaling and thus promoting sciatic nerve regeneration and functional recovery [93]. Additionally, exosomes derived from hypoxia-preconditioned MSCs improved cognitive decline through amelioration of synaptic dysfunction and regulation of inflammatory responses in APP/PS1 mice [41]. Hypoxia-treated MSCs were also found to overexpress antiapoptotic proteins like IL-8, IL-10, and Fas Ligand (FasL) and thus enhancing immunoregulation which in turn would inhibit inflammation and encourage tissue repair and regeneration [94].

### Preconditioning of MSCs with Pharmacological Agents

Preconditioning of MSCs with pharmacological agents is considered another effective way to enhance their actions [95, 96]. The preconditioning of MSCs has been examined through the use of pharmacological agents known for their neuroprotective and neurotrophic actions such as mood stabilizers like lithium and valproic acid [97]. Importantly, lithium and valproic acid control the signaling pathway mediating neurotrophic and neuroprotective functions through the inhibition of histone deacetylase (HDAC). In turn, the inhibition of HDAC prevents apoptosis and suppresses cellular glycogen synthase kinase-3 which mediates glycogen metabolism, cell proliferation and migration, and cellular transport [98]. Experimentally, deferoxamine preconditioning significantly increased total antioxidant, pro-angiogenic, neuroprotective, and anti-inflammatory factors secreted by MSCs and thus improving the therapeutic potential of adipose tissue-MSCs in DN [99]. The enhancement of exosome efficacy using different culture conditions in different nervous system diseases gives the hope of their possible efficacy in PN.

## Modified MSC-exos

Previous studies revealed different ways of MSC-exos modification to improve their therapeutic potential [100] through loading exosomal lumen with endogenous or exogenous biomolecules such as nucleic acids, peptides, or drugs or through modifying MSC-exos’ surface for targeting of a particular type of cells or tissues [101].

### Therapeutic Exosome Loading

MSC-exos have recently been introduced as promising biological carriers owing to their remarkable small size, biocompatibility, and ability to carry specific and various therapeutic molecules to their targeted cells [102]. The functional biomolecules in MSC-exos such as proteins and nucleotides are included in tissue regeneration, immunoregulation, and angiogenesis in various experimental models [103]. Exosome loading includes the addition of the therapeutic molecule to the exosome from its parent cell (endogenous loading) or direct depositing of the therapeutic molecule into purified exosomes (exogenous loading) [104].

As an example of endogenous loading, MSCs can be genetically modified to enhance the expression of endogenous biomolecules inside MSC-exos by using plasmids or viral vectors. Therefore, loading the targeted therapeutic biomolecules inside exosomes depends mainly on the genetic modifications of the parent cells for the improvement of the therapeutic actions of MSC-exos and prevention of heterogeneity between different exosome batches [103]. Exosomal miRNAs are also key regulators to their function. For example, treatment of DPN with MSC-exos enriched with miRNA-146a provided enhanced therapeutic efficacy in PN [74]. The administration of exosomes containing miR-133b also contributed to the inhibition of *ras* homolog gene family member A (RhoA) and activation of extracellular signal-regulating kinase 1/2 (ERK1/2) and cAMP response element-binding protein (CREB) pathway and thus amelioration of neuronal apoptosis and neurodegeneration in rats after intracerebral hemorrhage [105]. Besides, miR-133b-modified MSC-exos allowed miR-133b transfer to astrocytes and thus regulated their expression and promoted neuronal functional recovery after stroke in rats [106]. In addition, MSC-exos enriched with miR-126 and miR-26a enhanced the recovery of limb motor function and improved axon regeneration after SCI [107, 108]. Transplantation of engineered exosomes derived from BM-MSCs after being fused with polypyrrole nanoparticles containing liposomes improved DPN along with electrical stimulation. This approach normalized the velocity of nerve conduction and muscle action potential compared to the healthy control [75]. Notably, several research studies have revealed that MSC-exos could be used as drug carriers in addition to their traditional cargoes like miRNAs and peptides. The membrane structure of MSC-exos can preserve the biological activity and integrity of drugs in addition to possible achievement of targeted drug delivery through the modification of the membrane structure of MSC-exos [109]. For example, chemotherapeutic drugs (adriamycin and paclitaxel) were loaded into MSC-exos for the management of tumors with reduced drug side effects [110, 111]. In the future, MSC-exos can be used as carriers for potential drugs for the management of diabetes, cancer, and their related neuropathic complications.

### Surface Modification of Exosomes

Three common strategies are present for surface membrane modification of MSC-exos such as genetic engineering, chemical modification, and membrane fusion. Genetic engineering consolidates gene sequences of guided proteins or peptides with those of selected exosomal membrane proteins to effectively present specific peptides and proteins on exosomal surface and thus enhancing its targeting or function. On the other hand, chemical modifications reveal a variety of synthetic and natural receptors or ligands on the membrane surface of exosomes through noncovalent or covalent modifications. Finally, membrane fusion uses extrusion to combine exosomes with other membrane structures, an approach that confers new functional and therapeutic benefits to them [112]. For example, Yang et al. innovated a membrane-editing technique to confer the required functional membrane proteins into cellular membranes directly using a virus-mimetic fusogenic exosomes, enabling the exosomes to target specific tissues [113]. Another study used the fusion extrusion technique to modify MSC-exos with monocyte mimics to enhance their delivery to myocardial tissues with ischemic damage [114]. Collectively, the membrane fusion technique to modify MSC-exos opens new approaches for improvement of their cell-targeting therapeutic efficacy.

## Limitations of Current Studies and Future Directions

MSC-exos have been clearly recognized as the main regulators of the paracrine mechanism that promotes regeneration mediated by stem cells [115]. These nanoparticles reveal similar therapeutic effects to those exerted by MSCs and can be considered a powerful tool for cell-free–based therapy with several advantages compared to MSCs. Of note, the outcomes of use of MSC-exos in peripheral nerve injury are encouraging [114]. Although being safe and effective experimentally, this field needs further research and much work to fully determine the potential of MSC-exos clinically and to overcome the limitations of their application [116, 117].

MSC-exos are commonly isolated via differential or density-gradient ultracentrifugation [118]. Despite being the most cost-effective available method, ultracentrifugation is time-consuming, needs intensive work, and may yield impurities [119]. Other isolation methods include ultrafiltration, size exclusion chromatography, precipitation, and immune affinity capture [119] in addition to size-dependent and immunoaffinity-based microfluidic technologies [120]. Though being limited compared to the traditional methods, these new methods require relatively small amount of samples and can achieve high purity and rapid separation [119]. Limitations of MSC-exos isolation include also low yield under conventional culture conditions [121] in addition to low efficacy upon application after multiple passages in vitro due to MSC senescence [122]. Thus, improving the yield of MSC-exos and enhancing their efficacy or targeting ability are crucial through altering MSC cell culture conditions [123, 124] or modifying exosomes with different approaches [125]. Furthermore, concerns regarding changes in morphology and bioactivity of exosomes have been reported following long-term storage [126]. Cryopreservation using liquid nitrogen and other cryoprotective agents may overcome these issues [127]. Notably, the components of MSC-exos as well as their exact actions that mediate tissue repair and regeneration need to be fully investigated. There is also no guidance for the safety and the exact dose or amount of MSC-exos needed for treatment. More research is still required to select the best route of systemic administration of MSC-exos (intravenous, subcutaneous, or intramuscular routes) [128]. Collectively, the risks, safety, and challenges included in the use of MSC-exos need to be fully investigated before their clinical use in tissue repair and regenerative medicine.

## Conclusion

MSC-exos have received great attention recently as an emerging therapeutic agent for the management of PN because of their ability to suppress inflammation, regulate the immune system and angiogenesis, and promote tissue regeneration in a similar way to MSCs. Exosomes reveal several advantages, fewer side effects, and lower risks compared to their parent MSCs. Exosomes are relatively non-immunogenic and with very low tumorigenic potential. However, a series of challenges and difficulties need to be overcome to verify their purity, reproducibility, safety, biodistribution, clearance, and their molecular characteristics or contents. Furthermore, their long-term therapeutic effect is still unknown.

Importantly, future research is required to enhance the therapeutic efficacy of exosomes and to promote their clinical application where preconditioning of MSCs with hypoxia or pharmacological agents has been considered an effective approach to optimize the actions of their derived exosomes. The surface of exosomes can also be modulated for targeting of particular cell types. Moreover, exosomes can be loaded with targeted drugs, peptides, or genes with facilitated delivery due to their ability to cross biological barriers. Meanwhile, the signals and pathways involved in the actions of MSC-exos in the target cells and organs should be identified in detailed studies before being considered as a potential cell-free based therapy in PN.

## Data Availability

Not applicable

## Abbreviations

AMPK: AMP-activated protein kinase
BM-MSCs: Bone marrow mesenchymal stem cells
CEC-exos: Exosomes derived from cerebral endothelial cells
CIPN: Chemotherapy-induced peripheral neuropathy
CNPase: 2′,3′-cyclic nucleotide 3′-phosphodiesterase
CREB: cAMP response element-binding protein
DPN: Diabetic peripheral neuropathy
DSPN: Diabetic sensorimotor polyneuropathy
ERK1/2: Extracellular signal regulating kinase 1/2
GelMA: Gelatin-methacryloyl
HDAC: Histone deacetylase
HUCMSC: Human umbilical cord mesenchymal stem cell
Iba1: Ionized calcium binding adaptor molecule 1
IL: Interleukin
miRNAs: MicroRNAs
MSC-exos: MSC-derived exosomes
MSCs: Mesenchymal stem cells
mTOR: Mammalian target of rapamycin
NGCs: Nerve guidance channels
NF-κB: Nuclear factor kappa B
PN: Peripheral neuropathy
PCL: Polycaprolactone
PI3K: Phosphatidylinositol 3-kinase
PKB: Protein kinase B
rGO: Reduced graphene oxide
RhoA: *Ras* homolog gene family member A
SCI: Spinal cord injury
TLRs: Toll-like receptors
